# Silver Nanoparticle-Induced Phosphorylation of Histone H3 at Serine 10 Involves MAPK Pathways

**DOI:** 10.3390/biom9020078

**Published:** 2019-02-22

**Authors:** Xiaoxu Zhao, Yanying Rao, Jie Liang, Shoukai Lin, Xiumei Wang, Zhangliang Li, Jianhui Huang

**Affiliations:** 1 College of Environmental and Biological Engineering, Putian University, Putian 351100, China; yanyingrao@ptu.edu.cn (Y.R.); liangjie@ptu.edu.cn (J.L.); linshoukai@ptu.edu.cn (S.L.); wangxiumei@ptu.edu.cn (X.W.); lizhangliang@ptu.edu.cn (Z.L.); huangjh@ptu.edu.cn (J.H.); 2 Fujian Provincial Key Laboratory of Ecology-Toxicological Effects & Control for Emerging Contaminants, Putian 351100, China; 3 Key Laboratory of Ecological Environment and Information Atlas (Putian University) Fujian Provincial University, Putian 351100, China

**Keywords:** silver nanoparticles, phosphorylation, histone, mitogen-activated protein kinase (MAPK), pathway

## Abstract

The phosphorylation of histone H3 at serine 10 (p-H3S10) has been shown to be closely correlated with mitotic chromosome condensation. We previously reported that intracellular silver nanoparticles (AgNPs) release Ag ions that alter actin filament dynamics, leading to the activation of Aurora kinases and the formation of p-H3S10 through a mechanism clearly different from that occurring during mitosis. In the present study, we examined other mechanisms underlying the induction of p-H3S10 formation by AgNPs. We observed that the early formation of p-H3S10 induced by AgNPs occurred via the activation of mitogen-activated protein kinase (MAPK) pathways, specifically the Jun N-terminal protein kinase (JNK) and extracellular signal-regulated kinase (ERK) pathways. The late AgNP-induced p-H3S10 formation occurred via the activation of the entire MAPK cascade. On the other hand, p-H3S10 formation was not due to DNA damage induced by AgNPs, or the activation of the kinases ataxia telangiectasia-mutated (ATM) and ATM-Rad3-related (ATR). Several studies have compared the mechanism of AgNP toxicity to a Trojan horse-type molecular pathway. We observed different effects of AgNO_3_ (Ag^+^) and AgNPs on cells, and only the JNK inhibitor suppressed the temporary AgNO_3_-induced formation of p-H3S10. These results strongly indicate that AgNP-induced p-H3S10 formation does not rely solely on one signaling pathway, but rather may involve two or more pathways.

## 1. Introduction

Nanoparticles (NPs) (diameter of approximately 1–100 nm) have received considerable attention in recent decades due to their widespread applications in modern technology, health care, and commercial products [1]. Silver NPs (AgNPs) are the most widely used nanomaterial, and exhibit potent antibacterial and antifungal activities [2].

In the past, Ag has been regarded as a safe metal, and Ag products such as silverware have been used for a long time. However, the results of recent studies have indicated that exposure to AgNPs has several negative effects on biological systems. In vitro studies have demonstrated multiple undesirable physiological effects following exposure to AgNPs, such as the inhibition of cell proliferation, membrane damage, mitochondrial dysfunction, and apoptosis [3,4,5,6]. Because of their strong oxidative activity, AgNPs release silver ions that easily bind to the thiol groups of proteins in cells, resulting in cytotoxicity, genotoxicity, immunological responses, and even cell death [7,8,9,10]. The generation of reactive oxygen species (ROS) is another important mechanism underlying AgNP-induced toxicity, and the resultant oxidative stress may eventually injure the cell [3,4,5].

Among these types of damage, genotoxic effects have been regarded as a particularly important aspect of AgNP toxicity, as DNA damage can lead to mutations and potentially to the development of cancer and birth defects. DNA damage occurs when AgNPs directly interact with DNA or indirectly affect ROS generation. We recently showed that AgNPs induce DNA damage, which leads to phosphorylated histone H2AX (γ-H2AX) [11]. Mistakes during the repair of DNA damage, including DNA double-strand breaks, are an important factor in the development of genomic instability, which is closely associated with carcinogenesis [12]. Phosphorylated histone H2AX formation is regulated by the DNA damage response kinases: ataxia telangiectasia-mutated (ATM) and ATM-Rad3-related (ATR) [13].

Epigenetic dysregulation can also be induced by AgNPs, which may have long-term effects on gene expression reprogramming [14]. Silver NPs can affect the cell cycle and induce DNA hypermethylation through the p53 or p21 pathways, which may affect cells at an epigenomic level [14]. The results of our previous studies showed that AgNPs can significantly induce the phosphorylation of histone H3 at serine 10 (p-H3S10) [15]. Phosphorylated histone H3S10 is associated with mitotic chromosome condensation [16,17] and plays an important role in gene transcription associated with tumor promotion [18,19]. The induction of p-H3S10 formation can be enhanced by epidermal growth factor (EGF) and 12-*O*-tetradecanoylphorbol 13-acetate (TPA), which also promote carcinogenesis. Moreover, p-H3S10 formation can be induced by several environmental factors, such as cigarette side-stream smoke (CSS), ultraviolet B (UVB), arsenite, and nickel [20,21,22,23]. Arsenite-induced p-H3S10 formation was shown to be attributed to activation of the extracellular signal-regulated kinase (ERK) pathway in the mitogen-activated protein kinase (MAPK) cascade [20]. In addition, nickel was shown to promote H3S10 phosphorylation via the c-Jun N-terminal protein kinase (JNK) pathway [21], and CSS activates the JNK and Akt pathways, leading to p-H3S10 formation. Moreover, the induction of p-H3S10 formation by CSS, UVB, EGF, TPA, and arsenite has been associated with the expression of immediate-early genes, including the proto-oncogenes *c-fos* and *c-jun* [18,19,20,22,23]. This induction is regulated downstream of MAPK pathway activation. In recent studies, we demonstrated that AgNP-induced p-H3S10 formation is caused by abnormalities in actin polymerization and depolymerization upon cellular entry of AgNPs [24]. AgNPs incorporated into cells release Ag ions that alter the actin polymerization cycle. Dynamic changes in actin filaments activate Aurora kinases (AURKs) and induce p-H3S10 formation independent of the cell cycle. However, it was unclear whether the MAPK cascade and/or other signaling pathways mediate this process. Understanding the mechanism of AgNPs-induced p-H3S10 will be important for reducing the toxicity of AgNPs.

In the present study, we elucidated the mechanisms responsible for AgNP-induced p-H3S10 formation. We used several inhibitors to investigate the relationships between p-H3S10 formation and the MAPK and ATM/ATR pathways. The results revealed that AgNP-induced p-H3S10 formation is associated with two or more pathways.

## 2. Materials and Methods

### 2.1. Preparation of AgNPs

Silver NPs with a primary listed size of <0.1 µm were purchased from Sigma-Aldrich (St. Louis, MO, USA; cat. no. 576832) and were prepared as described previously [15]. Silver NPs were suspended in Dulbecco’s Modified Eagle’s Medium (DMEM; Thermo Scientific, Gaithersburg, MD, USA) containing 0.5% (*v*/*v*) fetal bovine serum (FBS; Life Technologies, Grand Island, NY, USA) at a final concentration of 10 mg/mL and were immediately sonicated in a bath-type sonicator (Bioruptor; Cosmo Bio, Tokyo, Japan) for 1 min before being applied to cells. The mean diameter of the AgNPs in DMEM was 425.9 nm [25].

### 2.2. Cells and Cell Culture Conditions

A potential route of exposure to AgNPs is through the respiratory system. In the present study, human lung adenocarcinoma cells (A549; provided by Shanghai Huiying Biological Technology Co., Ltd., Shanghai, China) were cultured in DMEM supplemented with 10% FBS and 100 U/mL penicillin-streptomycin at 37 °C in a humidified atmosphere containing 5% CO_2_. Adherent cell cultures were used in experiments during the logarithmic growth phase.

### 2.3. Treatment of Cells with AgNPs or Ag Ions

When the cells reached 70–80% confluence, the medium was changed to DMEM supplemented with 0.5% FBS. After being cultured for 24 h, the cells were treated with AgNPs (1 mg/mL) or AgNO_3_ (50 µM) for ~10 h. The cells were treated with formaldehyde (FA, 2 mM) for 2 h as a positive control.

In experiments on the inhibition of signaling pathways, the ERK inhibitor U0126 (10 µM), the JNK inhibitor SP600125 (10 µM) or the p38 inhibitor SB203580 (10 µM) were added 1 h before treatment with 1 mg/mL AgNPs or 50 µM AgNO_3_. Alternatively, the cells were treated with 1 mg/mL AgNPs for 7 h and then with U0126 (10 µM), SP600125 (10 µM), or SB203580 (10 µM) for 1 h. The inhibitors caffeine (5 mM), wortmannin (10 µM) and KU-55933 (10 µM) were added 0.5 h before treatment to inhibit the ATM/ATR pathway.

### 2.4. Western Blot Analysis

Cells treated with AgNPs or AgNO_3_ were lysed in lysis buffer and Western blotting was performed as described previously [15]. Primary antibodies against p-H3S10, γ-H2AX, phospho-ERK, ERK, phosphor-JNK, JNK, phosphor-p38, p38 (Cell Signaling Technology Inc., Danvers, MA, USA) (1:1000) were used, followed by secondary antibodies conjugated with horseradish peroxidase (Jackson Immuno Research Laboratories, West Grove, PA, USA) (1:1000).

## 3. Results

### 3.1. Induction of p-H3S10 Formation after Treatment with AgNPs Independent of DNA Damage

**Figure 1:**
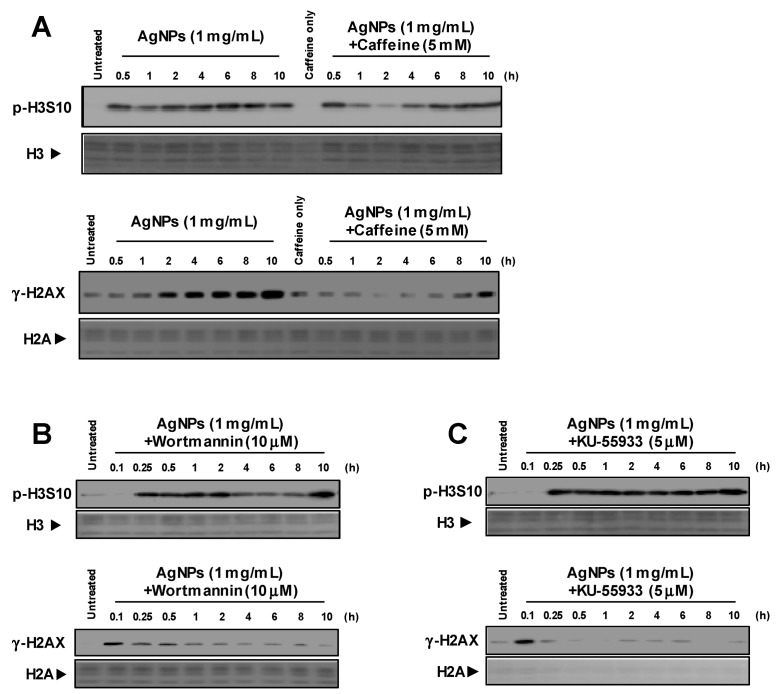
Generation of p-H3S10 after treatment with silver nanoparticles (AgNPs) independent of DNA damage. Different pathways responsible for the phosphorylation of H3S10 (p-H3S10) and H2AXS139 (γ-H2AX) were examined; (**A**) p-H3S10 and γ-H2AX formation in the presence of caffeine. A549 cells pretreated with caffeine (5 mM) for 0.5 h were then treated with AgNPs (1 mg/mL) for ~10 h; (**B**) p-H3S10 and γ-H2AX formation in the presence of wortmannin. A549 cells pretreated with wortmannin (10 µM) for 0.5 h were then treated with AgNPs (1 mg/mL) for ~10 h; (**C**) p-H3S10 and γ-H2AX formation in the presence of KU-55933. A549 cells pretreated with KU-55933 (10 µM) for 0.5 h were then treated with AgNPs (1 mg/mL) for ~10 h. H3 and H2A levels evaluated by Coomassie brilliant blue (CBB) staining were used as a standard for equal protein loading for SDS-PAGE.

We previously reported that AgNPs generate γ-H2AX, which occurs in part due to the production of intracellular oxidative products such as ROS [11]. Phosphorylated histone H2AX formation is regulated by the DNA damage response kinases ATM and ATR [13]. To elucidate the relationship between p-H3S10 formation and these kinases, cells were pretreated with caffeine, and an ATM and ATR inhibitor, prior to treatment with AgNPs. Phosphorylated histone H3S10 was generated in a time-dependent manner and was not suppressed by caffeine, as shown in Figure 1A. However, caffeine partially weakened the formation of γ-H2AX, which was mediated by treatment with AgNPs. Moreover, p-H3S10 formation was not affected by the presence of wortmannin or KU-55933, as shown in Figure 1B,C. Two inhibitors suppressed AgNP-induced γ-H2AX formation. These results suggested that p-H3S10 formation was not due to DNA damage induced by AgNPs or the activation of ATM/ATR. 

### 3.2. Histone H3S10 Was Phosphorylated via MAPK Pathways

**Figure 2:**
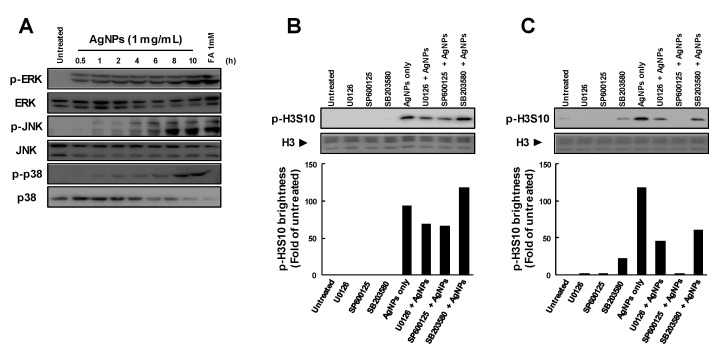
Relationship between mitogen-activated protein kinase (MAPK) activation and p-H3S10 formation after treatment with AgNPs; (**A**) time-dependent activation of MAPKs after treatment with AgNPs. A549 cells were treated with AgNPs (1 mg/mL) for ~10 h; (**B**) p-H3S10 formation in the presence of specific inhibitors of each MAPK pathway. A549 cells were pretreated with inhibitors (10 µM) for 1 h and then treated with AgNPs (1 mg/mL) for 1 h in the presence of the inhibitors. The p-H3S10 band intensity detected by Western blotting was assessed using an analysis tool in Adobe Photoshop CS5, and the ratio to the untreated control was plotted; (**C**) p-H3S10 formation in the presence of specific inhibitors of each MAPK pathway. A549 cells were pretreated with AgNPs (1 mg/mL) for 7 h and then treated with inhibitors (10 µM) for 1 h. The p-H3S10 band intensity detected by Western blotting was assessed using an analysis tool in Adobe Photoshop CS5 (Adobe Systems Inc, San Jose, CA, USA), and the ratio to the untreated control was plotted. H3 levels evaluated by CBB staining were used as a standard for equal protein loading for SDS-PAGE.

Mitogen-activated protein kinase cascades have been reported to be required for the formation of p-H3S10 induced by arsenite, UVB, EGF and other stimuli [12,20,26,27]. The results showed that AgNPs induced the phosphorylation of ERK, JNK and p38 in a time-dependent manner, as shown in Figure 2A. In cells pretreated with specific inhibitors before the addition of AgNPs, p-H3S10 formation at 1 h was slightly inhibited by the ERK inhibitor U0126 and the JNK inhibitor SP600125, as shown in Figure 2B, and was unaffected by the p38 inhibitor SB203580. Silver NPs-induced p-H3S10 was inhibited by U0126 or SP600125 in a dose-dependent manner, but the inhibitory effects of both inhibitors were nearly equal, as shown in the Supporting Information and Figure 1. Next, cells were treated with AgNPs, followed by the specific inhibitors, with the results showing that p-H3S10 formation at 7 h was slightly inhibited by U0126 and SB203580, and was significantly inhibited by SP600125, as shown in Figure 2C. A slight increase in p-H3S10 occurred after treatment with SB203580, but it was not significant. This may be because the mitosis-related p-H3S10 was not completely inhibited. These results indicated that the early AgNP-induced p-H3S10 formation occurred via activation of specific MAPK pathways, specifically the JNK and ERK pathways, while the later AgNP-induced p-H3S10 formation occurred via activation of the entire MAPK cascade.

### 3.3. Release of Ag Ions from AgNPs and MAPK Pathways

**Figure 3:**
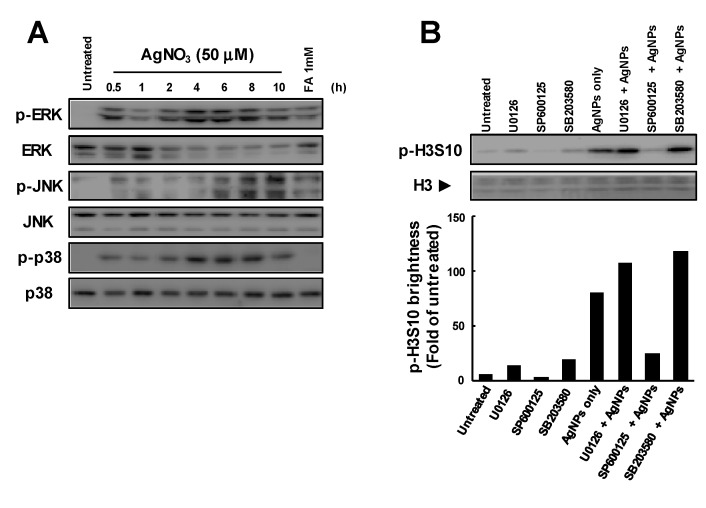
Relationship between MAPK activation and p-H3S10 formation after treatment with AgNO_3_; (**A**) Time-dependent activation of MAPKs after treatment with AgNO_3_. A549 cells were treated with AgNO_3_ (50 µM) for ~10 h; (**B**) p-H3S10 formation in the presence of specific inhibitors of each MAPK pathway. A549 cells were pretreated with inhibitors (10 µM) for 1 h and then treated with AgNO_3_ (50 µM) for 1 h in the presence of the inhibitors. The p-H3S10 band intensity detected by Western blotting was assessed using an analysis tool in Adobe Photoshop CS5, and the ratio to the untreated control was plotted. H3 levels evaluated by CBB staining were used as a standard for equal protein loading for SDS-PAGE.

We previously demonstrated that p-H3S10 formation was temporarily induced after treatment with AgNO_3_ but rapidly ceased. In this study, the phosphorylation of ERK, JNK and p38 was also induced after treatment with AgNO_3_, as seen in Figure 3A. The AgNO_3_-induced formation of p-H3S10 at 1 h was inhibited by SP600125 but not by U0126 or SB203580, as shown in Figure 3B. These results indicated that the early formation of p-H3S10 induced by Ag ions released from intracellular AgNPs occurred via the activation of MAPK pathways, primarily the JNK pathway. 

## 4. Discussion

In our previous studies, we described the rapid induction of p-H3S10 formation in cells treated with AgNPs, which was clearly different from that observed during mitosis [15,24]. Based on the rapid formation of p-H3S10 after treatment with AgNPs, the phosphorylation of H3S10 may have been induced via the activation of a signal transduction pathway. Signal transduction via the MAPK and AKT pathways has been reported to be involved in the effects of some carcinogenic substances [21,22,23]. Interestingly, p-H3S10 has been reported to play a role in the nucleosomal response downstream of the MAPK pathway activation in mammalian cells [22,28,29]. As AgNPs have been reported to activate MAPK pathways [30], the attenuation of p-H3S10 formation by MAPK inhibitors was expected to occur, and the results showed that the formation of p-H3S10 induced by AgNPs for 1 h was inhibited by U0126 and SP600125, but not by SB203580. Previous studies reported that Ag ions released from intracellular AgNPs alter actin filament dynamics, which causes the activation of AURKs and the induction of p-H3S10 formation [24]. These results strongly indicate that AgNP-induced p-H3S10 formation does not rely solely on one signaling pathway, but may involve two or more pathways at different treatment times, as shown in the Supplementary Materials and Figure 2. However, we observed a transient decrease in AgNP-induced p-H3S10 formation following treatment with caffeine at 2 h and wortmannin at 4~8 h, as shown in Figure 1B,C, but we do not know the reason for this effect. Several protein phosphatases, such as PP1, PP2A, and PP2B may also affect the AgNP-induced p-H3S10 [31]. This issue will be the subject of our next study.

Mitogen and stress-activated protein kinases 1 and 2 (MSK1 and MSK2) are activated by MAPKs (ERK, JNK or p38) and phosphorylate H3S10 and H3S28. Within the MAPK signaling pathways, phosphorylation is a response to a vast array of extracellular stimuli and stressors, including growth factors, UV light, alcohol, and metals [12,20,26,27]. Treatment with AgNPs results in the production of ROS, which generates γ-H2AX [11]. However, p-H3S10 formation in cells was not affected by caffeine, wortmannin, or KU-55933 treatments, as shown in Figure 1. These results indicate that the MAPK pathway activation leading to p-H3S10 by AgNPs is not caused by signal transduction from DNA damage.

Several studies have compared the mechanism of AgNP toxicity with a Trojan horse-type molecular pathway [32]. We observed a similar activation of MAPK after treatment with AgNO_3_, as seen in Figure 3; AgNO_3_-induced p-H3S10 formation was inhibited by a JNK inhibitor. In contrast to the findings for AgNPs, AgNO_3_-induced p-H3S10 formation was only inhibited by the JNK inhibitor. The amount of Ag ions released from AgNPs was approximately 0.1% (w/w) of the total amount of Ag loaded [33,34]. The amount of Ag ions released from AgNPs (1 mg/mL) was expected to be approximately 1 µg/mL (0.22 µM) during the 0.5 h incubation period. This concentration was much lower than the concentrations of AgNO_3_ used (50 µM). Ag ions released continuously from AgNPs incorporated into cells may be important for p-H3S10 formation. These results indicated that the lasting release of Ag ions from AgNPs inside cells might generate p-H3S10 for a long time after the initial uptake of AgNPs. On the other hand, the cellular uptake of AgNPs may differ from cell to cell and may involve diffusion, phagocytosis, and endocytosis [35]. In previous studies, we observed that p-H3S10 formation was temporarily induced by AgNO_3_ treatment but rapidly returned to baseline [15]. In this study, we showed that AgNP-induced p-H3S10 formation occurred via activation of the MAPK cascade, but p-H3S10 formation induced by AgNO_3_ occurred via the JNK pathway. This result may be due differences in how AgNPs and AgNO_3_ enter the cell. Further studies are needed to elucidate the mechanisms responsible for Ag ion-induced changes in p-H3S10 formation.

We summarized the relationship between AgNP-induced histone modifications and tumorigenic activity. AgNPs are considered tumor initiators because AgNP-induced γ-H2AX generation may result from oxidative DNA damage caused by increased intracellular oxidation [11]. On the other hand, AgNPs promote the phosphorylation of H3S10, which sits at the promoter regions of proto-oncogenes, thereby acting as tumor promoters [15]. These signals are primarily transmitted via the AURK pathways [24], with some contributions made from MAPK pathways.

## 5. Conclusions

The results of the present study describe a potential pathway for the induction of p-H3S10 formation by AgNPs. Intracellular AgNPs release Ag ions that activate MAPK and induce p-H3S10 formation independent of the ATM/ATR pathways. Future studies should focus on investigating the underlying mechanisms and the consequence of p-H3S10 formation in greater detail.

## Supplementary Materials

The following are available online at https://www.mdpi.com/2218-273X/9/2/78/s1, Figure S1: Dose-dependent activation of p-H3S10 formation in the presence of specific inhibitors of MAPK pathway, Figure S2: Potential mechanism for AgNP-induced p-H3S10.
