# Supplementary material for: Silver Nanoparticle-Induced Phosphorylation of Histone H3 at Serine 10 Involves MAPK Pathways
Source: Biomolecules. 2019 Feb 22;9(2):78. doi: 10.3390/biom9020078 (PMC6406294; doi:10.3390/biom9020078)
Supplement: Supplementary file 1 [file biomolecules-09-00078-s001.pdf]

Supplementary Material

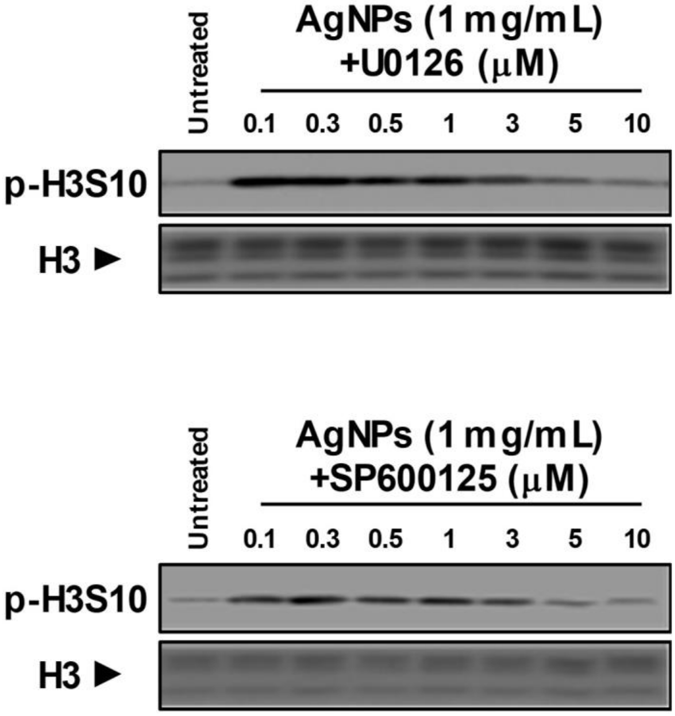

Figure S1. Dose-dependent activation of p-H3S10 formation in the presence of specific inhibitors of MAPK pathway. A549 cells were pretreated with U0126 or SP600125 (~ 10 μM) for 1 h and then treated with AgNPs (1 mg/mL) for 1 h in the presence of the inhibitors.

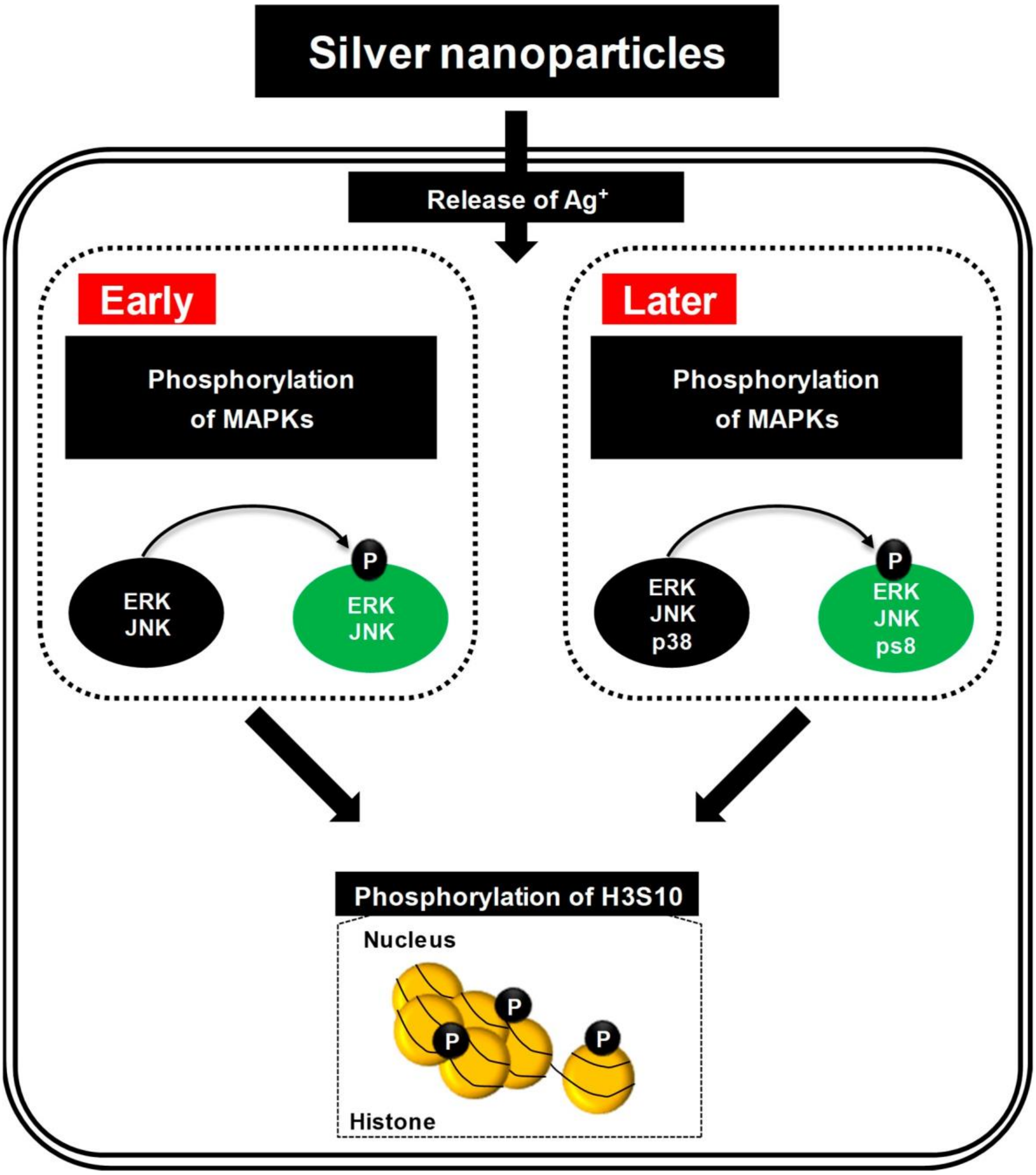

Figure S2. Potential mechanism for AgNP-induced p-H3S10 AgNPs incorporated into cells released Ag ions, which activated MAPK pathways and induced p-H3S10. The early AgNP-induced p-H3S10 formation occurred via activation of specific MAPK pathways, specifically the JNK and ERK pathways, while the later AgNP-induced p-H3S10 formation occurred via activation of the entire MAPK cascade.
